# Radon degassing triggered by tidal loading before an earthquake

**DOI:** 10.1038/s41598-021-83499-0

**Published:** 2021-02-18

**Authors:** Yasutaka Omori, Hiroyuki Nagahama, Yumi Yasuoka, Jun Muto

**Affiliations:** 1 Department of Radiation Physics and Chemistry, Fukushima Medical University, Fukushima, 960-1295 Japan; 2 Department of Earth Science, Graduate School of Science, Tohoku University, Sendai, 980-8578 Japan; 3 Institute of Radioisotope Research, Kobe Pharmaceutical University, Kobe, 658-8558 Japan

**Keywords:** Natural hazards, Solid Earth sciences, Geochemistry

## Abstract

The presence of anomalous geochemical changes related to earthquakes has been controversial despite widespread, long time challenges for earthquake prediction. Establishing a quantitative relationship among geochemical changes and geodetical and seismological changes can clarify their hidden connection. Here we determined the response of atmospheric radon (^222^Rn) to diurnal tidal (K1 constituent) loading in the reported 11-year-long variation in the atmospheric radon concentration, including its anomalous evolution for 2 months before the devastating 1995 Kobe earthquake in Japan. The response to the tidal loading had been identified for 5 years before the occurrence of the earthquake. Comparison between these radon responses relative to crustal strain revealed that the response efficiency for the diurnal K1 tide was larger than that for the earthquake by a factor of 21–33, implying the involvement of crustal fluid movement. The radon responses occurred when compressional crustal stress decreased or changed to extension. These findings suggest that changes in radon exhaled from the ground were induced by ascent flow of soil gas acting as a radon carrier and degassed from mantle-derived crustal fluid upwelling due to modulation of the crustal stress regime.

## Introduction

Radon is a radioactive noble gas, generated from radioactive decay of ^226^Ra in the ground, which migrates to the ground surface, and exhales into the atmosphere. It has been one of the most important, extensively studied geochemical elements for realising earthquake prediction since possible seismicity-related change in hot-spring radon concentration was first reported in 1927 in Japan^1^. Radon concentration changes have been observed in the groundwater, underground (soil and caves) air, and atmosphere for inland and thrust earthquakes and earthquake swarms^1–10^. However, despite the century-long, widespread challenges (see reviews^1–3^), seismicity-related radon changes, especially those before earthquake occurrences, remain questionable because most only claimed statistical existence of these changes. Only recently, have reproducible (periodic) patterns related to mechanical crustal loading (lake-water level and earth tide) been identified in radon time series^7,8,11–14^. To clarify hidden physical mechanisms connecting crustal deformation from earthquakes and radon changes, a quantitative relationship should be established between crustal strain and radon changes.

**Figure 1:**
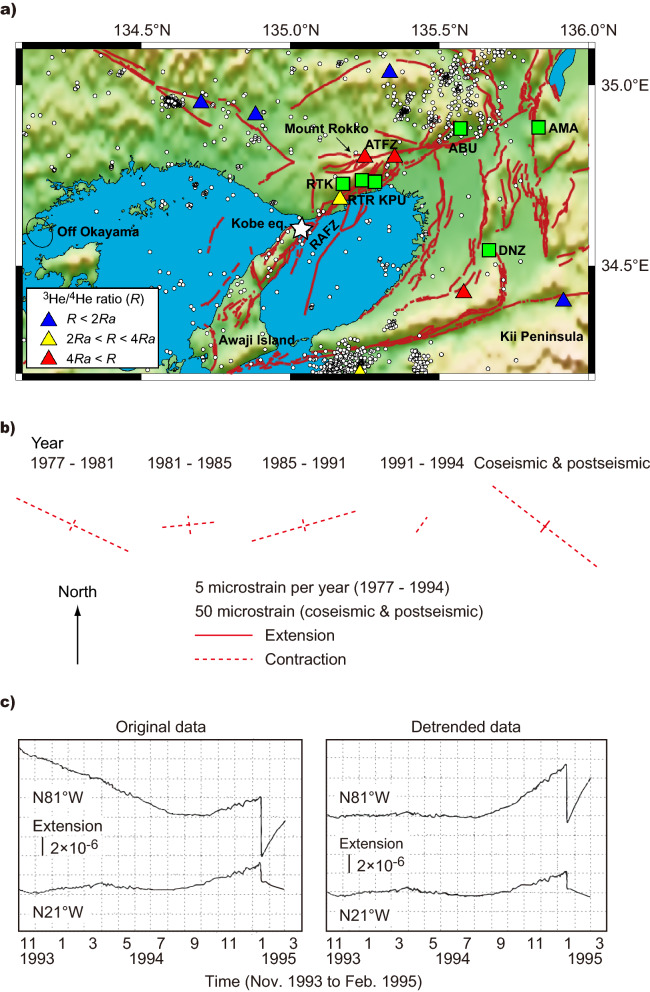
Geological and tectonic characteristics of the Kobe earthquake occurrence area. (**a**) Distributions of epicentres of seismicity from 1 January 1984 to 16 January 1995 (magnitude ≥ 2.0, depth ≤ 20 km; open circles) and the Kobe earthquake (star), measurement sites of atmospheric radon concentration and crustal strain (green squares), those of helium isotopes in mineral and hot springs (triangles), and active faults (red lines). The triangles are coloured depending on the corrected ^3^He/^4^He ratio normalised by the value (*R*_*a*_: 1.39 × 10^–6^) for the air (see the inset). Underneath the western area depicted by an open ellipse, the subducting Philippine Sea Plate might be locked during 1991–1993 (ref.^40^). *ABU* Abuyama, *AMA* Amagase, *DNZ* Donzurubou, *KPU* Kobe Pharmaceutical University, *RTK* Rokko–Takao, *RTR* Rokko–Tsurukabuto, *ATFZ* Arima–Takatsuki fault zone, *RAFZ* Rokko–Awaji fault zone. Data on seismicity were obtained from the Unified Japan Meteorological Agency Earthquake Catalogue (http://evrrss.eri.u-tokyo.ac.jp/tseis/jma1/index.html) and those on topography, helium isotopes, and active faults were obtained from refs.^51,17,16^, respectively. (**b**) Variations in principal crustal strain rates observed at RTR from 1977 to 1995 (Ref.^21^). (**c**) Time-series variations in crustal strain observed at RTK from November 1993 to February 1995 (Ref.^19^). The map was created using the Generic Mapping Tools^52^.

Here, the crustal strain–radon change relationship combined with cases of tectonically induced changes by earth tides and an earthquake was investigated. This study focused on the atmospheric radon concentration anomaly preceding the Kobe earthquake (17 January 1995; moment magnitude 6.9), the second-most disastrous earthquake in Japan in the past 30 years. The geological and tectonic characteristics of this earthquake occurrence area were E–W oriented compressional stress field, dense distribution of E–W to NE–SW oriented strike-slip active faults (e.g. the Rokko–Awaji fault zone (RAFZ) and Arima–Takatsuki fault zone) with large dip angles of 60°–90°, and upwelling of deep crustal mantle-derived fluids with high ^3^He/^4^He ratios^15–18^ (Fig. 1). This earthquake followed several-years-long to several-months-long precursory anomalies in geophysical, hydrological, and geochemical components (background seismic activities, crustal strain, groundwater discharge, and groundwater radon/chloride ion)^6,19–26^ (Table 1). For instance, the crustal strain field began to vary during 1990–1991: E–W contractional strain decreased in the RAFZ^21^ (Fig. 1b), whereas N–S oriented contractional strain increased in the east from the RAFZ^25^ (Table 1). Subsequently, the contractional strain field changed to extensional from mid-1994 to the earthquake occurrence in the study area^19,25^ (Fig. 1c; Table 1). Among the reported geophysical to geochemical precursory changes, Yasuoka and Shinogi^26^ observed anomalous increases in atmospheric radon concentration for 2 months prior to the earthquake. The increase was attributed to elevated radon exhalation by a factor of up to 5 compared to the normal amount of radon exhalation during October 1984 to January 1994^27^, which was the result^28^ of a response to crustal strain change in the order of 10^–8^–10^–6^. Based on this observation it is proposed that the radon concentration likely responds to tidal loading and transient crustal strain change in the order of 10^–8^. Successful extraction of radon response to tidal loading is direct, reliable evidence for the reproducible connection between the geophysical and geochemical phenomena, that clarifies the physical mechanism of precursory geochemical phenomena before earthquakes.

**Table 1:** Occurrence of geophysical, hydrological, and geochemical precursors before the Kobe earthquake (17 January 1995).

Component	Location	Occurrence times	Phenomena	References
**Intermediate-term precursor**				
Elevation	RTK	1991 to 1994	Elevation uplifted	^19^
Crustal strain	RTK	1992 to 1994	Amplitude and phase of O1 constituent changed	^24^
	RTR	1991 to 1994	Contraction decreased	^21^
	ABU, AMA, DON	1990 to late 1992	N–S oriented contraction increased	^25^
Seismicity	RAFZ	From 1990	Seismic quiescence occurred	^22^
	34–36° N; 134–136° E	1991 to 1993 From 1994	*b* value increased *b* value decreased	^23^
	Off Okayama	1991 to 1993	Background seismicity decreased	^40^^a^
Atmospheric radon	KPU	1990 to 1994	Response to tide (K1) occurred	This study
**Short-term precursor**				
Crustal strain	RTK	From Sep. 1994	Crustal strain changed to extension	^19^
	ABU, AMA, DON	From mid-1994	N–S oriented strain changed to extension	^25^
Groundwater discharge	RTK	From Nov. 1994	Discharge rate increased with an intermittent peak	^19^
Atmospheric radon	KPU	From Nov. 1994	Concentration increased above the normal level	^26^
Groundwater chloride ion	Rokko	From Aug. 1994	Concentration increased with a peak occurring after the mainshock	^20^
Groundwater radon	Nishinomiya^b^	From Oct. 1994 to 9 Jan. 1995	Concentration increased with intermittent peaks	^6^

^a^Ide and Tanaka^40^ did not report the decrease in background seismicity as a precursor.

^b^Nishinomiya is located 30 km northeast of the mainshock epicentre.

## Results and discussion

**Figure 2:**
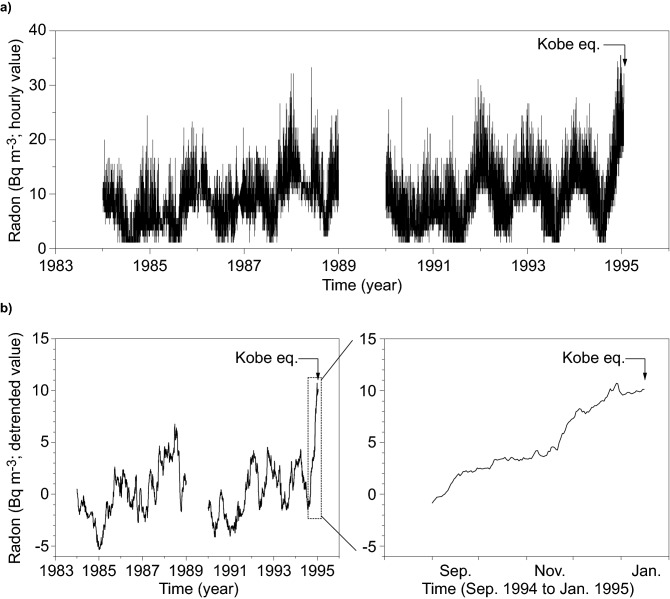
Time-series variations in atmospheric radon concentration observed at KPU. (**a**) Time-series variation drawn using hourly original data. (**b**) Time-series variation drawn using detrended data of smoothed daily minimum radon concentrations. The smoothed daily minimum values were analysed because of those less sensitive to daily variations in meteorological factors (e.g., wind speed, thermal stabilisation).

Atmospheric radon concentration was monitored hourly between 1984 and 1995 except for a period in 1989 at the Kobe Pharmaceutical University (KPU). The monitoring site was located on the RAFZ striking along the foot of Mount Rokko, approximately 27 km north–east from the epicentre (Fig. 1a). Figure 2a shows an 11-year variation in atmospheric radon concentration measured there. The radon concentration increased from September 1994, and subsequently, exceeded the normal variation range 2 months prior to the earthquake, which could be viewed as a precursory change^26^. The detrended data showed that the radon concentration change reached up to 10 Bq m^−3^ (Fig. 2b). Meanwhile, the crustal strain also changed from contractional to extensional, measured at the Rokko–Takao (RTK) Observatory situated in Mount Rokko^19^ (Fig. 1c). The detrended data showed that the change in the areal extensional strain reached up to 5.2 × 10^–6^. A coincidence of these changes indicates that the increased radon exhalation from the ground was caused by crustal deformation preceding the earthquake^28^. Based on these findings, as a measure of the sensitivity of radon to crustal deformation, the ratio of radon (*ΔC*_e_) to areal strain (*Δε*_e_) change related to the earthquake was determined to be 1.9 × 10^6^ Bq m^−3^, considering a radon change of 10 Bq m^−3^ associated with an areal crustal strain change of 5.2 × 10^–6^.

**Figure 3:**
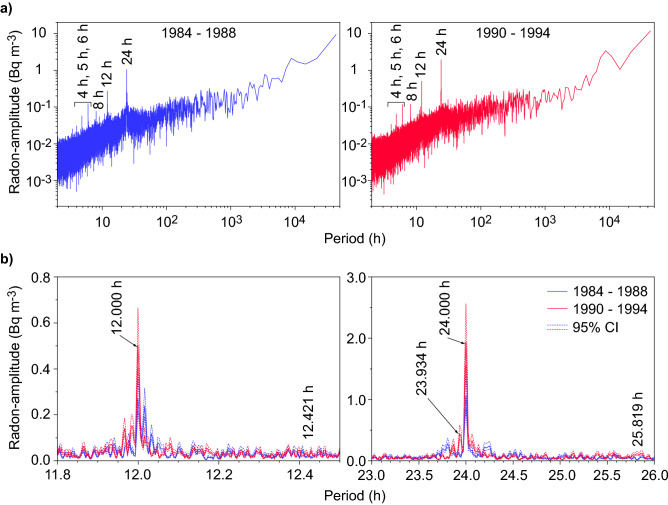
Amplitude spectra for hourly time series in atmospheric radon concentration. The 1984–1988 dataset and 1990–1994 dataset were analysed separately. (**a**) Amplitude spectra for whole periods. (**b**) Amplitude spectra around the periods of 12 h (left panel) and 24 h (right panel).

For the two sets of hourly continuous radon data in Fig. 2a, fast Fourier transform (FFT) analysis was applied to investigate the radon response to periodic crustal strain change induced by tidal loading. Dominant tidal loading cycles have diurnal, luni-solar (K1), and lunar (O1) constituents, and semidiurnal, principal solar (S2), and principal lunar (M2) constituents (Table 2). Their relation in amplitude-magnitude is M2 > K1 > O1 > S2 in Kobe. Figure 3a shows the amplitude spectra for the radon datasets in 1984–1988 and 1990–1994. A few sharp peaks were identified clearly at ≤ 24-h cycles in both datasets, most of which were related to meteorological factors^29^.

**Table 2:** Tidal constituents in the study area and radon signals.

Tide				Radon signal		Remarks
Constituent	Symbol	Period (h)	Amplitude rank	1984–1988	1990–1994	
Lunar	O1	25.819	3	None	None	
Solar	S1	24.000	> 4	Exist	Exist	Meteorological factors
Luni-solar	K1	23.934	2	None	Exist	
Principal lunar	M2	12.421	1	None	None	
Principal solar	S2	12.000	4	Exist	Exist	Meteorological factors

The amplitude spectra around the targeting tidal cycles are enlarged in Fig. 3b. In the shorter-cycle range, no sharp peaks were identified at the cycle corresponding to the M2 constituent. Although a sharp peak was identified at the 12.000-h cycle, it is attributed to meteorological factors (e.g., air pressure and wind speed) rather than the S2 constituent because, despite the larger M2 tidal loading than the S2 loading in the study area, the 12.421-h cycle was not seen in the radon time series.

In contrast, significantly different spectra were observed in the 1984–1988 and 1990–1994 datasets for the longer-cycle range. Only one sharp peak was observed at the 24.000-h cycle for the 1984–1988 dataset, whereas a weak, but clear peak was also identified at the 23.934-h cycle as well as 24.000-h cycle for the 1990–1994 dataset. The radon change at the 24.000-h cycle was attributed to thermal stabilisation/destabilisation in the atmospheric boundary layer driven by solar radiation^30^ rather than the solar S1 tidal loading because of no peak at the 25.819-h cycle induced by the larger O1 constituent. However, the 23.934-h cycle was not induced by any meteorological factors, which is confirmed by no corresponding cycle peak for the 1984–1988 dataset. Accordingly, the 23.934-h cycle of radon change was induced by K1 tidal loading. This is the first evidence of periodic release of geochemical gas from the ground to the atmosphere driven by periodic crustal loading. The amplitude spectrum revealed that the radon concentration change reached up to 0.44 Bq m^−3^ (95% confidence interval: 0.36–0.59 Bq m^−3^). It should be noted that no information regarding the radon response to the earth tides was given in 1989 when the radon monitoring was unavailable.

The ratio of radon concentration (*ΔC*_t_) to areal strain (*Δε*_t_) change related to the K1 tidal loading was evaluated further; the areal strain induced by solid earth and ocean tide loading was calculated theoretically using the GOTIC2 software (ver. 2004.10.25) developed by the National Astronomical Observatory, Japan^31^. The calculated areal strain by the K1 tidal loading was 0.93 × 10^–8^ based on the RTK site locality information. Thus, the ratio (*ΔC*_t_/*Δε*_t_) was evaluated as 47 × 10^6^ Bq m^−3^ (95% confidence interval: (39–63) × 10^6^ Bq m^−3^) considering radon changes of 0.44 Bq m^−3^ (95% confidence interval: 0.36–0.59 Bq m^−3^) associated with an areal crustal strain change of 0.93 × 10^–8^ by the K1 tide. The values were larger than the *ΔC*_e_/*Δε*_e_ value for the case preceding the 1995 Kobe earthquake by a factor range of 21–33.

Two potential mechanisms can be considered to induce the response of atmospheric radon to crustal deformation. One qualifier is the development of crustal rock microfractures due to applied loading. Microfracture development would increase (1) radon production rate due to increased rock grain surface area and (2) radon transport pathways (i.e., permeability) due to connections between isolated microcracks^32,33^, which would consequently elevate radon exhalation. However, microfracture development is an irreversible process; therefore, it cannot reproduce the periodic pattern induced by the tidal loading. In addition, radon that originates deeper cannot reach the ground surface because it decays with a mean lifetime of 5.5 days. Additionally, assuming a soil porosity of 0.4, effective diffusion coefficient of 10^–6^ m^2^ s^−1^, and subsurface ascent air flow velocity of 10^–6^ m s^−1^, the diffusion and advection lengths of radon are estimated to be 0.7 and 1.2 m in soil, respectively^34,35^. It implies that the exhaled radon is not produced in the basement rocks, but only in the uppermost soil layer. Therefore, microfracture development in crustal rocks is an unlikely primary physical mechanism for current radon exhalation responding to crustal deformation.

The other potential mechanism is a change in subsurface vertical flow of air carrying the radon. The air flow modulates the vertical soil radon profile; consequently, radon is exhaled from the ground surface without a change in radon production rate by newly forming microfractures. King^4^ reported profile changes in soil radon concentration responding to an earthquake with magnitude 4.0 on the San Andreas Fault system (USA). At the San Jacinto Fault (USA), Birchard and Libby^36^ observed increases and decreases in soil radon concentrations in the quadrants of seismic compression and dilatation, respectively, following an earthquake. These results could be qualitatively explained by the induction of subsurface ascending or descending air flow from a change in loading stress. Here, for several months prior to the 1995 Kobe earthquake, the increase in atmospheric radon concentration corresponded to an increase in the groundwater discharge rate at the RTK site and changes in the crustal strain from widespread contraction to extension, namely at RTK, Abuyama (ABU), Amagase (AMG), and Donzurubou (DNZ) sites^19,25^. Unlike the case of Birchard and Libby^36^, which indicated a phenomenon of squeezing out soil gas through soil-pore volume compression, the current case indicated that crustal fluids upwelled due to subsurface pathway expansion, and radon carriers degassed from the fluids induced subsurface ascent air flow. In the study area, non-volcanic mantle-derived fluids upwelled at hot springs and mineral springs^17,18,37^. Umeda et al.^37^ noted that the NW–SE oriented extensional stress field generating open fractures favoured upwelling of mantle-derived fluids in the area. The fluids contained abundant quantities of gases such as carbon dioxide that can carry radon toward the earth’s surface. Similar association of extensional tectonics elevating carbon dioxide degassing from the deep crust into the atmosphere was recently recognised worldwide^38,39^. Tamburello et al.^39^ also noted an important role of normal or strike-slip faults with large dip angles on carbon dioxide degassing in addition to extensional tectonics. Combined with the tectonic settings in the study area, induced subsurface ascent air flow due to mantle-derived fluid upwelling is likely a physical mechanism of radon exhalation responding to crustal deformation.

Questions arise as to why, unlike 1984–1988, the radon exhalation responding to the K1 tidal loading occurred in 1990–1994 and why its sensitivity was high compared to the case of earthquake occurrence. We propose that initiation of the tidal radon response can be regarded as a precursory phenomenon and it is caused by upwelling deep crustal fluids. During the latter period, precursory phenomena were determined from the geodetic and seismologic observations (elevation, crustal strain, and seismic activity; Fig. 1b,c, Table 1). These observations indicated that the crustal stress regime change occurred almost synchronously in the broad area surrounding the epicentral region. The timing also coincided with the tidal radon response occurrence, although it is unclear whether the response was present in 1989 when radon monitoring was unavailable.

The coincidence of occurrence is probably explained by deep crustal fluids upwelling and reaching the RAFZ where the stress state favoured upwelling. East of the RAFZ (ABU, AMA, and DNZ), Morii et al.^25^ suggested that the stress regime change was caused by plate boundary contact beneath the eastern Kii Peninsula. Similarly, off Okayama, west of the RAFZ, quiescence of background seismicity occurred and positive correlation between the numbers of background seismicity and deep tremors predicted by the tide disappeared from 1991 to 1993^40^, which implied plate boundary contact beneath the area. However, unlike the stress state around the RAFZ, compressional stress decreased at RTR, and elevation uplift and tide phase and amplitude modulation in crustal strain occurred at RTK of the RAFZ^19,21,24^. The locally different stress fields could be explained by a change in pore pressure related to the upwelling of deep crustal fluids. They reached the RAFZ and increased pore pressure around 1990, leading to RTK site uplift as well as compressional stress field reduction.

The stress field changes from fluid upwelling could also initiate the radon response to diurnal tide variation. The upwelled fluids would increase pore pressure and reduce effective stress on the fault plane, which consequently generates the response to the small diurnal tide variation magnitude. The stress-induced change in the tidal crustal gas discharge response was evident from the reports by Weinlich et al.^7,8^ demonstrating disturbances in diurnal cycles of radon and carbon dioxide discharges induced by earth tides due to crustal stress redistribution following earthquake swarms in the Eger Rift, Czech Republic. The dynamic stress change induced by the passage of seismic waves originating from the Izmit earthquake (magnitude 7.4) in 1999 caused an apparent modification of the spectrum of confined-well water radon concentration and the disappearance of the tidal (M2) signal in the radon time-series^3^. In connection with the role of fluids, recent laboratory triaxial creep experiments for quartz-rich sandstone samples revealed that pore-pressure oscillation reduced effective stress and caused cyclic deformation (acoustic emission events) of the samples^41,42^. In the present study area, the water injection experiment conducted at the Nojima Fault on Awaji Island, the southern part of the RAFZ following the Kobe earthquake showed that water injection into the fault amplified the diurnal crustal strain cycle produced by the earth tide^43,44^. Therefore, the diurnal tide phase and amplitude modulation in crustal strain records at RTK, observed as an intermediate-term precursor before the earthquake (Table 1), were possibly affected by external fluid injection^24^, that is, deep crustal fluid upwelling into the RAFZ. The amount of degassing from the fluids was controlled and amplified by the tidal loading thereby initiating the atmospheric radon response to the diurnal tide. The deep crustal fluid contribution was supported from the present study finding that the radon sensitivity (*ΔC*_t_/*Δε*_t_) to tidal high-frequency strain change was larger than that (*ΔC*_e_/*Δε*_e_) to the monotonic (i.e., low-frequency) strain change during 4 months before the earthquake, implying involvement of crustal fluid movement.

Some previous studies^45,46^ did not consider changes in radon concentration related to earth tides. This is likely because a peak corresponding to the strongest M2 tide could not be identified from spectral analysis. However, we determined the weak peak of radon at 23.934 h as the earth-tide (K1) signal, as in previous studies by Crockett et al.^47^, Groves-Kirkby et al.^13^ and Weinlich et al.^8^ who reported signals induced by weaker earth tides. There are two possible reasons for the radon signal at the M2 tidal period being absent. From a meteorological viewpoint, the atmospheric radon concentration reported herein was primarily controlled by the variation in atmospheric stability due to the 24-h solar cycle. The night-time thermal stabilisation attained in the atmospheric boundary layer leads to the accumulation of radon exhaled from the crust, whereas daytime thermal destabilization disperses accumulated and exhaled radon immediately via convection^29,30,48^. Such a strong meteorological effect would possibly mask a radon signal related to the M2 tide, which is stronger in tidal force but higher in frequency than the K1 tide. From a tectonic viewpoint, modulation of the crustal stress regime preceding the earthquake may cause signal amplification at a specific period. As described above, in concordance with the crustal stress modulation, only diurnal variations in crustal strain were altered at the site along the RAFZ^24^. A similar phenomenon was observed in the water injection experiment conducted after the earthquake^43,44^. In addition, the deformation of quartz-rich sandstone samples is dependent on the frequency of pore-pressure oscillation rather than its amplitude^49^; samples exposed to lower-frequency pore-pressure oscillation exhibited substantial deformation. Accordingly, it can be considered that modulation of the crustal stress regime during 1990–1994 amplified only the radon signal related to the K1 tide.

We presented the two cases of precursory changes in atmospheric radon concentration associated with the crustal strain changes preceding the earthquake. The crustal strain–radon change relationships apparently had different radon sensitivities to crustal strain for the earth tide and earthquake. The sensitivity of radon to crustal deformation has been only surficially examined, even including the present case, so that further observations are required to generalise their quantitative relationship and clarify the physical mechanism behind it. Multi-station observations are also important to confirm the precursory changes in atmospheric radon concentration. Such observations could not be made in the present study because the work was performed at KPU only. After the earthquake, we established a nation-wide network for the measurement of atmospheric radon concentration by radioisotope facilities throughout Japan^50^, which could be used to confirm the validity of identified precursory changes preceding future earthquakes.

The atmospheric radon variation reported here contained several-years-long and several-months-long precursory changes associated with the 1995 Kobe earthquake governed by the compression to extension stress regime change. Additionally, the stress regime changes should accompany the discharge of radon carriers like carbon dioxide, indicating upwelling of non-volcanic mantle-derived fluids through the strike-slip active faults with large dip angles as identified and characterised in the study area. These two characteristic processes constrained the geogenic radon variation before the earthquake.

## Methods

### Area description

The study area is in a high-strain-rate field (> 0.1 × 10^–6^ year^−1^) corresponding to the western margin of the Niigata–Kobe Tectonic Zone^53^. Right-lateral active faults are distributed striking NE–SW (Rokko–Awaji fault zone; RAFZ) and ENE–WSW (Arima–Takatsuki fault zone; ATFZ)^16^. The Kobe earthquake (on 17 January 1995) initiated at north of Awaji Island and ruptured the RAFZ north-eastward. Mount Rokko, composed of granite and granodiorite, is behind the centre of Kobe City. Kobe Pharmaceutical University (KPU), the observation station for atmospheric radon concentration, was located at the foot of Mount Rokko, whereas the underground Rokko–Takao (RTK)^19^ and Rokko–Tsurukabuto (RTR)^21^ observatories for crustal strain were located inside Mount Rokko at 240 and 100 m in depth, respectively. In the eastern part of the study area, the underground observatories were also located at Abuyama, Amagase, and Donzurubou (ABU, AMA, and DNZ)^25^.

Several-years-long to several-months-long geophysical and hydrological precursors were determined as well as atmospheric radon concentration before the Kobe earthquake. For several-years-long geophysical precursors, the elevation was uplifted by 1.5 mm inside the RTK observatory for 5 years before the earthquake^19^. At the same site, phase and amplitude changes had been observed in the O1 constituent of a laser strainmeter record since 1992^24^. The contractional strain rate estimated by extensometer records changed from ENE–WSW to NE–SW and decreased in magnitude from 1991 to 1994 at the RTR site^21^ (Fig. 1b). Along the RAFZ, seismic quiescence had occurred since 1990 for background seismic activity on the RAFZ^22^. In the east of the study area, N–S components of strain records showed an increase in contraction during 1990 to 1992 at the ABU, AMA, and DNZ sites^25^. At off Okayama, west of the RAFZ, background seismicity appeared to decrease during 1991–1993 although Ide and Tanaka^40^ did not note it. In wide area (34–36° N; 134–136° E) including the present study area, the *b* value^23^ increased from 1991 to 1993, and then it decreased in 1994 for seismic activities with magnitude > 2. For several-months-long geophysical and hydrological precursors, N–S oriented crustal strain changed to extension after mid 1994 at the ABU, AMA, and DNZ sites^25^. Similarly, at the RTK site, crustal strain measured by borehole strain meters switched from contraction to extension from September 1994 (Fig. 1c), which accompanied a gradual increase in groundwater discharge rate from November 1994 with an intermittent peak triggered by the external force of a distant earthquake^19^. The reported precursory observations, including geochemical precursors^6,20,26^, are briefly summarised in Table 1.

### Radon monitoring

Atmospheric radon concentration was measured hourly using a flow-type ionisation chamber (Model NAG513, Fuji Electric Systems Co., Ltd, Japan) with a large detection volume of 1.8 × 10^–2^ m^3^. Air at 5 m above the ground was sampled at a flow rate of 15.6 m^3^ min^−1^, and a part of the filtered air was introduced into the ionisation chamber at a flow rate of 6.7 × 10^–3^ m^3^ min^−1^. Continuous monitoring occurred between 1984 and 1995 except for 1989 due to system maintenance. Details can be found in Yasuoka and Shinogi^26^.

### Periodic analysis

FFT was applied to the two radon datasets (1984–1988 and 1990–1994) to derive the amplitude spectra. Welch’s method^54^ was adopted to reduce estimation uncertainty (i.e., 95% confidence interval). In the 1984–1988 time-series, the first 32,768 (= 2^15^) data were analysed by FFT with the Hamming window function to obtain its power spectrum density. Subsequently, another 32,768 data shifted from the first data by 720 data (approximately 1 month) forward were analysed to obtain the second power spectrum density. This procedure was repeated until the analysis of the whole 1984–1988 time-series was completed. Then, based on the obtained power spectrum densities, the average power spectrum density with 95% confidence interval was calculated and it was converted into an amplitude spectrum. The same procedure was applied to the 1990–1994 time-series to obtain its amplitude spectrum. In the periodic analysis, the software Signal Processing Toolbox of MATLAB ver. R2018b (the MathWorks, USA) was used. The frequency resolution of the spectral analysis was 0.000143 h^−1^. This is equal to approximately 0.013 h of time resolution at the 24.0-h period. In addition, the spectral leakage, as a ratio of the amplitude at the 23.934-h period to that at the 24.000-h period, was determined to be 0.005.

## Data Availability

Figure 1a was created using data on seismicity, topography, active fault distribution, and helium isotope ratio. The Unified Japan Meteorological Agency Earthquake Catalogue is available at: http://evrrss.eri.u-tokyo.ac.jp/tseis/jma1/index.html. The topographic data^51^ are available at: https://www.ngdc.noaa.gov/mgg/global/. Active fault distribution^16^ is available at: https://gbank.gsj.jp/activefault/index_e_gmap.html. Data on the helium isotope ratio were obtained from Sano and Wakita^17^. Figure 1b,c are from refs.^21,19^, respectively. The data on atmospheric radon that support the findings of this study are available from the corresponding author upon reasonable request.

## Code availability

The FFT analysis was made using the software Signal Processing Toolbox of MATLAB ver. R2018b (the MathWorks, USA). Areal strain induced by the earth and ocean tides was evaluated using the software GOTIC2 (ver. 2004.10.25)^31^. Figure 1a was created using the Generic Mapping Tools^52^. Figures 2 and 3 were drawn using the software OriginPro ver. 2020 (OriginLab Corporation, USA).

## References

[1] King C-Y (1986). Gas geochemistry applied to earthquake prediction: An overview. J. Geophys. Res..

[2] Cicerone RD, Ebel JE, Britton J (2009). A systematic compilation of earthquake precursors. Tectonophysics.

[3] Woith H (2015). Radon earthquake precursor: A short review. Eur. Phys. J. Spec. Top..

[4] King C-Y (1978). Radon emanation on San Andreas Fault. Nature.

[5] Wakita H, Nakamura Y, Notsu K, Noguchi M, Asada T (1980). Radon anomaly: A possible precursor of the 1978 Izu-Oshima-kinkai earthquake. Science.

[6] Igarashi G (1995). Ground-water radon anomaly before the Kobe earthquake in Japan. Science.

[7] Weinlich FH (2006). Seismically induced variations in Mariánské Lázně fault gas composition in the NW Bohemian swarm quake region, Czech Republic—a continuous gas monitoring. Tectonophysics.

[8] Weinlich FH, Stejskal V, Teschner M, Poggenburg J (2013). Geodynamic processes in the NW Bohemian swarm earthquake region, Czech Republic, identified by continuous gas monitoring. Geofluids.

[9] Oh YH, Kim G (2015). A radon-thoron isotope pair as a reliable earthquake precursor. Sci. Rep..

[10] Iwata D, Nagahama H, Muto J, Yasuoka Y (2018). Non-parametric detection of atmospheric radon concentration anomalies related to earthquakes. Sci. Rep..

[11] Trique M, Richon P, Perrier F, Avouac JP, Sabroux JC (1999). Radon emanation and electric potential variations associated with transient deformation near reservoir lakes. Nature.

[12] Aumento F (2002). Radon tides on an active volcanic island: Terceira, Azores. Geofis. Int..

[13] Groves-Kirkby CJ, Denman AR, Crockett RGM, Phillips PS, Gillmore GK (2006). Identification of tidal and climatic influences within domestic radon time-series from Northamptonshire, UK. Sci. Total Environ..

[14] Richon P, Moreau L, Sabroux J-C, Pili E, Salaün A (2012). Evidence of both M_2_ and O_1_ Earth tide waves in radon-222 air concentration measured in a subglacial laboratory. J. Geophys. Res..

[15] Katao H, Maeda N, Hiramatsu Y, Iio Y, Nakao S (1997). Detailed mapping of focal mechanisms in/around the 1995 Hyogo-ken Nanbu earthquake rupture zone. J. Phys. Earth.

[16] National Institute of Advanced Industrial Science and Technology. *Active Fault Database of Japan, August 11, 2015 Version* (Geological Survey of Japan, National Institute of Advanced Industrial Science and Technology, 2015). https://gbank.gsj.jp/activefault/index_e_gmap.html.

[17] Sano Y, Wakita H (1985). Geographical distribution of ^3^He/^4^He ratios in Japan: Implications for arc tectonics and incipient magmatism. J. Geophys. Res..

[18] Sano Y, Nakajima J (2008). Geographical distribution of ^3^He/^4^He ratios and seismic tomography in Japan. Geochem. J..

[19] Kyoto University and the University of Tokyo (1995). Observations of crustal movements and discharge change at Rokko-Takao station. Rep. Coord. Comm. Earthq. Predict..

[20] Tsunogai U, Wakita H (1995). Precursory chemical changes in ground water: Kobe Earthquake, Japan. Science.

[21] Kyoto University (1996). Strain changes observed at Rokko-Tsurukabuto station. Rep. Coord. Comm. Earthq. Predict..

[22] Watanabe H (1998). The 1995 Hyogoken-Nanbu earthquake and the accompanying seismic activity—behavior of the background seismicity. Annu. Disas. Prev. Res. Inst. Kyoto Univ..

[23] Enescu B, Ito K (2001). Some premonitory phenomena of the 1995 Hyogo-Ken Nanbu (Kobe) earthquake: Seismicity, b-value and fractal dimension. Tectonophysics.

[24] Hirose I, Kawasaki I, Takemoto S, Tamura Y (2003). Temporal variations of tidal constituents in strainmeter records prior to the occurrence of two large earthquakes. J. Geod. Soc. Jpn..

[25] Morii W (2006). Unusual change of the crustal strain preceding the 1995 Hyogoken-nanbu earthquake. Annu. Disas. Prev. Res. Inst. Kyoto Univ..

[26] Yasuoka Y, Shinogi M (1997). Anomaly in atmospheric radon concentration: A possible precursor of the 1995 Kobe, Japan, earthquake. Health Phys..

[27] Yasuoka Y (2012). Anomalous change in atmospheric radon concentration sourced from broad crustal deformation: A case study of the 1995 Kobe earthquake. Appl. Geochem..

[28] Yasuoka Y (2009). Preseismic changes in atmospheric radon concentration and crustal strain. Phys. Chem. Earth.

[29] Galmarini S (2006). One year of ^222^Rn concentration in the atmospheric surface layer. Atom. Chem. Phys..

[30] Omori Y (2009). Variation of atmospheric radon concentration with bimodal seasonality. Radiat. Meas..

[31] Matsumoto K, Sato T, Takanezawa T, Ooe M (2001). GOTIC2: A program for computation of oceanic tidal loading effect. J. Geod. Soc. Jpn..

[32] Koike K, Yoshinaga T, Suetsugu K, Kashiwaya K, Asaue H (2015). Controls on radon emission from granite as evidenced by compression testing to failure. Geophys. J. Int..

[33] Girault F, Schubnel A, Pili É (2017). Transient radon signals driven by fluid pressure pulse, micro-crack closure, and failure during granite deformation experiments. Earth Planet. Sci. Lett..

[34] Clements WE, Wilkening MH (1974). Atmospheric pressure effects on ^222^Rn transport across the Earth-air interface. J. Geophys. Res..

[35] Nazaroff WW (1992). Radon transport from soil to air. Rev. Geophys..

[36] Birchard GF, Libby WF (1980). Soil radon concentration changes preceding and following four magnitude 4.2–4.7 earthquakes on the San Jacinto Fault in southern California. J. Geophys. Res..

[37] Umeda K, Kanazawa S, Kakuta C, Asamori K, Oikawa T (2006). Variations in the ^3^He/^4^He ratios of hot springs on Shikoku Island, southwest Japan. Geochem. Geophys. Geosyst..

[38] Lee H (2016). Massive and prolonged deep carbon emissions associated with continental rifting. Nat. Geosci..

[39] Tamburello G, Pondrelli S, Chiodini G, Rouwet D (2018). Global-scale control of extensional tectonics on CO_2_ earth degassing. Nat. Comm..

[40] Ide S, Tanaka Y (2014). Controls on plate motion by oscillating tidal stress: Evidence from deep tremors in western Japan. Geophys. Res. Lett..

[41] Chanard K (2019). Sensitivity of acoustic emission triggering to small pore pressure cycling perturbations during brittle creep. Geophys. Res. Lett..

[42] Noël C, Passelègue FX, Giorgetti C, Violay M (2019). Fault reactivation during fluid pressure oscillations: Transition from stable to unstable slip. J. Geophys. Res. Solid Earth.

[43] Fujimori K (2001). Strain and tilt changes measured during a water injection experiment at the Nojima Fault zone, Japan. Is. Arc.

[44] Mukai A, Fujimori K, Ishii H, Nakao S (2001). Strain changes due to water injection experiments. Earth Mon. (Gekkan Chikyu).

[45] Mentes G (2018). Investigation of the relationship between rock strain and radon concentration in the tidal frequency-range. J. Appl. Geophys..

[46] Barberio MD (2018). Diurnal and semidiurnal cyclicity of radon (^222^Rn) in groundwater, Giardino Spring, central Apennines, Italy. Water.

[47] Crockett RGM, Gillmore GK, Phillips PS, Denman AR, Groves-Kirkby CJ (2006). Tidal synchronicity of built-environment radon levels in the UK. Geophys. Res. Lett..

[48] Omori Y, Nagahama H (2016). Radon as an indicator of nocturnal atmospheric stability: A simplified theoretical approach. Boundary-Layer Meteorol..

[49] Noël C, Pimienta L, Violay M (2019). Time-dependent deformations of sandstone during pore fluid pressure oscillations: Implications for natural and induced seismicity. J. Geophys. Res. Solid Earth.

[50] Yasuoka Y, Nagahama H, Muto J, Mukai T (2018). The anomaly in atmospheric radon concentrations prior to the 2011 Tohoku-Oki earthquake in Japan. Radiat. Environ. Med..

[51] Amante C, Eakins BW (2009). ETOPO1 1 Arc-minute Global Relief Model: Procedures, Data Sources and Analysis. NOAA Technical Memorandum NESDIS NGDC-24.

[52] Wessel P, Smith WHF, Scharroo R, Luis J, Wobbe F (2013). Generic mapping tools: Improved version released. EOS Trans. AGU.

[53] Sagiya T, Miyazaki S, Tada T (2000). Continuous GPS array and present-day crustal deformation of Japan. Pure Appl. Geophys..

[54] Welch PD (1967). The use of fast Fourier transform for the estimation of power spectra: A method based on time averaging over short, modified periodograms. IEEE Trans. Audio Electroacoust..

